# Supplementary material for: Adapting a medication adherence app for adolescents and young adults in Makurdi, Benue State: The Lu Dedoo Project
Source: PLoS One. 2026 May 29;21(5):e0350279. doi: 10.1371/journal.pone.0350279 (PMC13221014; doi:10.1371/journal.pone.0350279)
Supplement: Attachment — Submitted filename: Response to Reviewers.docx [file pone.0350279.s001.docx]

**Manuscript ID:** PONE-D-25-66140
**Title:** *Adapting a Medication Adherence App for Adolescents and Young Adults in Makurdi, Benue State: The Lu Dedoo Project*

Dear Dr. Jahun and Reviewers,

**Response to Reviewers**

Thank you for the careful review of our manuscript and for the constructive comments. We appreciate the opportunity to revise the manuscript. We have carefully All changes have been incorporated into both the tracked and clean versions of the manuscript.

Below, we provide a point-by-point response.

Response to the Academic Editor

**Comment 1: Qualitative methodology requires more detail**
“The section is lacking several details such as language used in FGD, were the questionnaires in English or local languages, were the translated? Were field notes collected. How was the transcription done from audio to text? How many transcripts were initially coded to develop the coding framework? Please provide the coding framework as figure or describe it explicitly. How was inductive and deductive coding approaches applied? E.t.c.”

**Response:**
Thank you for this important comment. We have substantially revised the qualitative methodology section to improve transparency and reproducibility. Specifically, we have now clarified that all FGDs were conducted in English; the discussion guide was developed and administered in English and was not translated; field notes were collected during each discussion; all FGDs were audio-recorded and transcribed verbatim by a trained research assistant; transcript accuracy was checked against the audio files by a second trained research assistant together with the first author; and three transcripts were initially coded to develop the preliminary coding framework. We have also explicitly described the combined deductive and inductive coding approach and added a coding framework table.

**Comment 2: Participant numbers and group details are unclear**
“It is unclear about the number of participants. Please provide demographic characteristics of the participants. Also provide details of each group participating in the FGD.”

**Response:**
Thank you. We have revised the manuscript to clearly report the final achieved FGD sample and group composition. The revised manuscript now states that a total of **22 participants** took part in three FGDs: one mixed-gender adolescent group aged 15–19 years (**n = 6**), one male young adult group aged 20–24 years (**n = 8**), and one female young adult group aged 20–24 years (**n = 8**).

**Comment 3: Funding information should not appear in Acknowledgments**
“It was stated that ‘This work was supported by EDCTP2 (EDCTP TMA2019SFP-2812’, is this the funder? Funding information shouldn’t appear under acknowledgement.”

**Response:**
Thank you. We have removed all funding-related text from the Acknowledgements section. Funding information is now presented only in the Funding Statement, in accordance with PLOS ONE requirements.

**Response to Reviewer 1**

**Comment 1: Recruitment wording is inconsistent**

**Comment:**
“Regarding participants recruitment refer to line 119 provider referral and line 149 consecutive recruitment”

**Response:**
Thank you for noting this inconsistency. We have revised the manuscript to harmonise the description of recruitment. Participants were identified during routine clinic visits with support from clinic staff and were then enrolled consecutively from among those who met the eligibility criteria and agreed to participate.

**Comment 2: Number of study sites is inconsistent**
“Regarding number of study sites refer to line 119 study was conducted at three HIV treatment clinics versus line 150 two study sites”

**Response:**
Thank you. We have corrected this inconsistency in the revised manuscript. The study setting and participant recruitment sections now consistently reflect the actual qualitative study sites used for the FGDs. The study was done in 3 facilities.

**Comment 3: Transport stipend wording**
“Line 155 revise transport stipend of USD 10 equivalent in local currency”

**Response:**
Thank you. We have revised this wording in the manuscript to improve clarity and local relevance. The revised text now states the amount received in Naira

**Response to Reviewer 2**

**Comment 1**
“The author should clearly define what MASI stands for at first mention.”

**Response:**
Thank you. We have revised the manuscript to define MASI in full at first mention as Masakhane Siphucule Impilo Yethu.

**Comment 2: Participants should be selected based on documented non-adherence to validate effectiveness**

**Comment:**
“Participants should be selected based on documented non-adherence in order to properly validate the effectiveness of the app.”

**Response:**
Thank you for this thoughtful comment. We respectfully clarify that the present manuscript reports the formative qualitative adaptation phase of the Lu Dedoo project rather than the effectiveness phase. The purpose of this manuscript is to describe how the MASI app was contextually adapted for adolescents and young adults living with HIV in Benue State using user-centered qualitative input. It is therefore focused on usability, acceptability, and adaptation needs rather than validation of clinical effectiveness.

**Comment 3: Inferential statistical analysis should compare adherence levels using viral load results before and after intervention**

**Comment:**
“Inferential statistical analysis should be used to compare adherence levels, using viral load results before and after the intervention.”

**Response:**
Thank you. We agree that such analyses are important for evaluating effectiveness. However, these analyses fall within the subsequent pilot randomized component of the larger study and are outside the scope of the present manuscript, which is focused on the formative qualitative adaptation phase.

**Comment 4: Follow newly enrolled clients for seven months and stable clients for one year for viral load assessment**

**Comment:**
“The study should follow newly enrolled clients for seven months and stable clients for one year to allow for meaningful viral load assessment.

**Response:**
Thank you for this suggestion. We agree that viral load follow-up duration is an important consideration for the later effectiveness study. However, this recommendation pertains to the pilot trial phase and not to the current qualitative adaptation manuscript. We have therefore not introduced viral load outcome analysis into this paper.

**Comment 5: A focus group discussion should be incorporated alongside quantitative analysis**

**Comment:**
“A focus group discussion (FGD) should be incorporated alongside the quantitative analysis to capture participants’ perspectives and experiences.”

**Response:**
Thank you. We agree with this point, and indeed, the current manuscript is centred on the FGD component of the study. The present paper specifically reports on the formative focus group phase conducted prior to the pilot trial to capture participants’ perspectives, app-use experiences, and needs for contextual adaptation.

**Comment 6: It is not possible to conclude that the app improves adherence without supporting clinical data**
“Overall, it is not possible to conclude that the app improves adherence among AYALHIV without supporting clinical data to provide objective evidence.”

**Response:**
Thank you. the current manuscript is centred on the FGD component of the study. The present paper specifically reports on the formative focus group phase conducted prior to the pilot trial to capture participants’ perspectives, app-use experiences, and needs for contextual adaptation of the app.

**Response to Journal Requirements**

**Requirement 1: PLOS ONE style requirements**

**Response:**
We have revised the manuscript to align with PLOS ONE formatting and style requirements and will submit the files using the required naming convention.

**Requirement 2: Funding statement must include all support and state no additional external funding**

**Response:**
We have prepared an amended Funding Statement for the cover letter and submission system:

**Funding Statement:**
“This work was supported by the European & Developing Countries Clinical Trials Partnership 2 (EDCTP2) programme under grant number EDCTP TMA2019SFP-2812, through the Desmond Tutu Health Foundation, South Africa. There was no additional external funding received for this study.”

**Requirement 3: Expand EDCTP2 in full**

**Response:**
We have expanded EDCTP2 in full in the Funding Statement and cover letter as **European & Developing Countries Clinical Trials Partnership 2 (EDCTP2)**.

**Requirement 4: Remove funding text from the manuscript body/Acknowledgments**

**Response:**
We have removed funding-related text from the Acknowledgments section and retained funding information only in the Funding Statement. The editor letter specifically noted the prior Acknowledgments wording.

**Requirement 5: Data Availability Statement should include repository/access details**

**Response:**
We have revised the Data Availability Statement to provide the repository name and access details for the de-identified dataset.

**Requirement 6: Ethics statement should only appear in the Methods section**

**Response:**
We have moved the ethics statement so that it appears only in the Methods section and removed it from any other section of the manuscript.

**Requirement 7: Copyrighted figures**

**Response:**
We have deleted Figures 1, 2, and 3

**Requirement 8: Tables 1, 2, and 3 must be cited in text**

**Response:**
We have revised the Results section to ensure that Tables 1, 2, and 3 are explicitly referred to in the text.

**Requirement 9: Clarify consent for interview transcript publication and de-identification**

**Response:**
We confirm that the manuscript includes only de-identified excerpts from focus group discussions and not full raw transcripts. All quotations were reviewed to ensure that they do not contain names, initials, dates, addresses, contact details, ID numbers, or other direct identifiers.

We thank the Academic Editor and both reviewers for their careful assessment and constructive suggestions. We believe the revised manuscript is stronger, clearer, and more fully aligned with the qualitative scope of the present study.

Sincerely,

Dr Olaposi Olatoregun

On behalf of all authors
